# Establishment of a novel ER-stress induced myopia model in mice

**DOI:** 10.1186/s40662-023-00361-2

**Published:** 2023-11-01

**Authors:** Longdan Kang, Shin-ichi Ikeda, Yajing Yang, Heonuk Jeong, Junhan Chen, Yan Zhang, Kazuno Negishi, Kazuo Tsubota, Toshihide Kurihara

**Affiliations:** 1 Laboratory of Photobiology, Keio University School of Medicine, 35 Shinanomachi, Shinjuku-Ku, Tokyo, 160-8582 Japan; 2 Department of Ophthalmology, The First Hospital of China Medical University, Shenyang, 110001 China; 3 Department of Ophthalmology, Keio University School of Medicine, 35 Shinanomachi, Shinjuku-Ku, Tokyo, 160-8582 Japan; 4 Tsubota Laboratory, Inc., 34 Shinanomachi, Shinjuku-Ku, Tokyo, 160-0016 Japan

**Keywords:** Tunicamycin, Myopia, ER stress, Myopia model

## Abstract

**Background:**

Recent studies have indicated a strong correlation between endoplasmic reticulum (ER) stress and myopia and that eyedrops containing the ER stress inducer tunicamycin (Tm) can induce myopic changes in C57BL/6 J mice. Therefore, this study aimed to create a new myopia model using Tm eyedrops and to explore the mechanism of ER stress-mediated myopia development.

**Methods:**

Three-week-old C57BL/6 J mice were treated with different concentrations (0, 25, 50, and 100 μg/mL) and/or number of applications (zero, one, three, and seven) of Tm eyedrops. Refraction and axial length (AL) were measured before and one week after Tm treatment. Scleral collagen alterations were evaluated under polarised light after picrosirius red staining. ER stress-related indicators, such as the expression of collagen I and cleaved collagen were detected using Western blotting.

**Results:**

Compared with the control group, mice administered eyedrops with 50 μg/mL Tm only once showed the greatest myopic shifts in refraction and AL elongation and reduced scleral expression of collagen I. Picrosirius red staining showed a lower percentage of bundled collagen in the Tm group. Expression of ER-stress indicators increased in the Tm groups. Furthermore, optimised administration of Tm induced matrix metalloproteinase-2 (MMP2) expression in the sclera, which plays a major role in collagen degradation.

**Conclusions:**

We have demonstrated that ER stress in the sclera is involved in myopia progression. Tm eyedrops induced myopic changes, loosening of the scleral collagen and decreased expression of collagen I. This process may be associated with ER stress in the sclera, which upregulates the expression of MMP2 leading to collagen degradation.

**Supplementary Information:**

The online version contains supplementary material available at 10.1186/s40662-023-00361-2.

## Background

Myopia, a global disease, affects a vast majority of the population, especially teenagers. In the next 20 to 30 years, the global prevalence of myopia and high myopia is expected to increase to 50% and 10%, respectively [1]. Therefore, a stable and reliable animal model is essential for the study of myopia. The most common animal models of myopia are form-deprivation myopia (FDM) and lens-induced myopia (LIM) [2–4]. Recently, our team found that mice with LIM showed endoplasmic reticulum (ER) stress in the sclera, which is similar to the results of other researchers using the FDM model [5, 6]. Furthermore, we previously showed that scleral ER stress causes myopia [5, 6]. This clearly indicates a correlation between myopia and ER stress. Therefore, based on the findings in our previous study, we sought to establish a reliable animal model of myopia by inducing ER stress in the scleral cells.

As a classic ER stress inducer, tunicamycin (Tm) is a nucleoside antibiotic that is isolated from the fermentation broths of *Streptomyces lysosuperficus* and *Streptomyces chartreusis* [7]. It potently induces ER stress by inhibiting the N-linked glycosylation of glycoproteins in the ER of eukaryotic cells, and thus disrupts protein maturation [8]. Remodelling of the scleral extracellular matrix (ECM) is a major feature of eye growth and myopia which mainly involves excessive degradation and reduced synthesis of the scleral ECM [9]. The sclera is mainly composed of collagen and its major function is to stabilize the size and shape of the eye, and structural alterations in collagen have also been observed in the scleral tissue of humans with high myopia [10, 11]. Therefore, scleral collagen crosslinking has been proposed as a therapeutic treatment to improve the scleral rigidity to inhibit eye growth in progressive myopia [12, 13].

Therefore, in this study, we explored a stable myopia model using Tm eyedrops and found morphological alterations in the scleral collagen. Notably, the model in this study simulated, to a limited extent, the scleral collagen changes that occur during the natural myopia process.

## Methods

### Mice and eyedrop treatment

All animal experiments in this study were approved by the Animal Experimental Committee of Keio University (permit number: 16017). Our study adhered to the Institutional Guidelines on Animal Experimentation at Keio University, the ARVO Statement for the Use of Animals in Ophthalmic and Vision Research, and Animal Research: Reporting of In Vivo Experiments (ARRIVE) guidelines for the use of animals in research. We purchased male C57BL/6 J mice (three weeks old) from CLEA Japan (Yokohama, Japan). Five mice were maintained in one cage with free intake of standard chow and water. We kept them in an environment with a 12 h/12 h light/dark cycle (the dark cycle extended from 8:00 p.m. to 8:00 a.m.) at 23 ± 3 °C.

According to the manufacturer’s instructions, Tm (Cayman Chemical, Catalogue #:11445) was dissolved in dimethyl sulfoxide (DMSO) at a concentration of 100 mg/mL, stored at − 20 °C, and diluted 1000-fold with phosphate-buffered saline (PBS) immediately before use to prepare eyedrop solutions of 25, 50, and 100 μg/mL. Mice were treated with different concentrations and/or a different number of applications of Tm eyedrops, and PBS containing the same concentration of DMSO was used as the control, 5 μL per eye. Ocular components were measured, and scleral tissues were collected for the assays on days 0, 7 and 21 after treatment. The experimental plan for the animals is shown in Additional file 1: Fig. S1.

### Ocular components measurement

AL was measured from the anterior corneal surface to the retinal pigment epithelium using a spectral domain optical coherence tomography (SD-OCT) system (Envisu R4310, Leica, Germany). The refraction was detected using an eccentric infrared photo refractor (Steinbeis Transfer Center, Germany) at the vertical pupil meridian. The data were automatically recorded by the program when the parameter values were stable. Choroidal thickness (ChT) was measured using SD-OCT, and the posterior surface of the choroid was quantified using Image J (Ver 1.53, NIH). ChT was determined using the formula: area divided by circumference. Intraocular pressure (IOP) was measured using a tonometer (Tono Lab, Icare Finland Oy, Vantaa, Finland) calibrated for mice under anaesthesia. Parts images of ocular components measurement were shown in Additional file 5: Fig. S5.

Mice were anesthetized with 0.75 mg/kg medetomidine (Sandoz K.K., Tokyo, Japan), 4 mg/kg midazolam (Domitor®, Orion Corporation, Espoo, Finland), and 5 mg/kg butorphanol tartrate (Meiji Seika Pharma Co., Ltd., Tokyo, Japan) dissolved in normal saline. Mydriasis was induced by 0.5% tropicamide eye drops (Santen, Osaka, Japan), and mice were placed in a cylindrical holder for measurement.

### Paraffin sections, analysis of collagen and TUNEL assay

The eyeballs were harvested after euthanising the mice and fixed in Super Fix (KURABO, Osaka, Japan) for three days at 4 °C with gentle shaking. The fixative solution was changed every day. After fixation, paraffin sections were prepared as previously described [6]. To measure collagen content, paraffin sections were stained using a picrosirius red stain kit (Polysciences, Inc., USA) according to the manufacturer’s instructions. In brief, sections were de-paraffinized and hydrated with deionised water (DI) and then placed into different solutions in the following order: solution A (phosphomolybdic acid) for 2 min, followed by rinsing with DI water; solution B for at least 60 min; and solution C for 2 min. Finally, the sections were dehydrated and cleared using 70% ethanol for 45 s. The sections were visualised under bright field and polarised light (Olympus BX53). Collagen shows birefringence under polarised light, and picrosirius red staining can demonstrate both bundled and unbundled collagen fibres. All reconstructed images were converted to 8-bit using ImageJ (v.1.53, NIH). A threshold was set to distinguish and analyse collagen size and the size of collagen fibres. Collagen fibres with sizes above the threshold were considered bundled collagen fibres. It is worth noting that the threshold value remains the same for all images analysed. Pixel intensity was measured thereafter (Additional file 1: Fig. S4).

TUNEL assay was performed using an in situ Apoptosis Detection Kit following the manufacturer’s protocol (TAKARA Bio, Shiga, Japan) and images were captured with a BZ-X800 system (Keyence, Tokyo, Japan).

### Sample preparation

After experimental intervention and measurement of ocular parameters, eyeballs were enucleated from C57BL/6 J mice. For protein expression analysis, isolated cornea, lens, retina, choroid and sclerae from eyeballs were homogenized in RIPA buffer (50 mM HEPES (pH 7.5), 150 mM NaCl, 1% NP-40, 0.1% sodium deoxycholate, 1 mM EDTA, 5 mM benzamidine, 10 mM β-glycerophosphate, 1 mM Na_3_VO_4_, 50 mM NaF, and 1 mM PMSF) containing Halt protease inhibitor cocktail (ThermoFisher Scientific, Waltham, MA, USA). After centrifugation, protein concentration was measured using the BCA (Pierce BCA Protein Assay Kit, Thermoscientific, Waltham, MA, USA) method and adjusted to 1.0 g/L with Laemmli sample buffer (Nacalai Tesque, Kyoto, Japan). Samples were stored at − 30 °C until further use.

### Western blotting

To visualize protein expression, sodium dodecyl sulphate–polyacrylamide gel electrophoresis was performed using 10% acrylamide gels with protein-size markers (MagicMark XP Western Protein Standard, ThermoFisher Scientific). Proteins were transferred to polyvinylidene fluoride membranes (Merck Millipore, MA, USA), blocked with Blocking One (Nacalai Tesque, Tokyo, Japan), and incubated overnight at 4 °C with the following antibodies: anti-ATF6 (24169-1-AP, Proteintech, CHI, USA), IRE1 alpha (phosphor Ser724) (GTX132808, GeneTex, CA, USA), anti-col1-3/4 mAb (0217-050, Immunoglobe, Germany), COL1A1 Rabbit mAb (#84336), IRE1 (14C10) Rabbit mAb (#3294), phosphor-PERK (Thr980, 16F8) Rabbit mAb (#3179), PERK (C33E10) Rabbit mAb (#3192), MMP2 Rabbit mAb (#4022), Bip (C50B12) Rabbit mAb (#3177), phosphor-eIF2α (Ser51) (D9G8) XP Rabbit mAb (#3398), eIF2α (D7D3) XP Rabbit mAb (#5324),and β-actin (8H10D10) Mouse mAb (#3700) (Cell Signaling Technologies Japan, Tokyo, Japan). Membranes were incubated with the appropriate horseradish peroxidase-conjugated secondary antibodies and visualised using SuperSignal West Femto Maximum Substrate (ThermoFisher Scientific, Waltham, MA, USA). After visualisation, the membranes were incubated with Restore PLUS Western Blot Stripping Buffer (ThermoFisher Scientific, Waltham, MA, USA) and re-incubated with another primary antibody. Results for phosphorylated and total, and target and β-actin antibody stripping used the same membrane. Densitometric analyses were performed using ImageJ software (v.1.53, NIH). Uncropped blots in this manuscript were shown in Additional file 3: Fig. S3.

### Statistical analysis

Differences between the control and experimental groups were compared using Student’s t-test or One-way analysis of variance with least significant difference (LSD) post hoc test to calculate statistical significance (GraphPad Prism software, v.8.0). All data are expressed as mean ± SD. Statistical significance was set at *P* < 0.05.

## Results

### Tm (50 μg/mL) induced a significant myopic change in C57BL/6 J mice

**Fig. 1:**
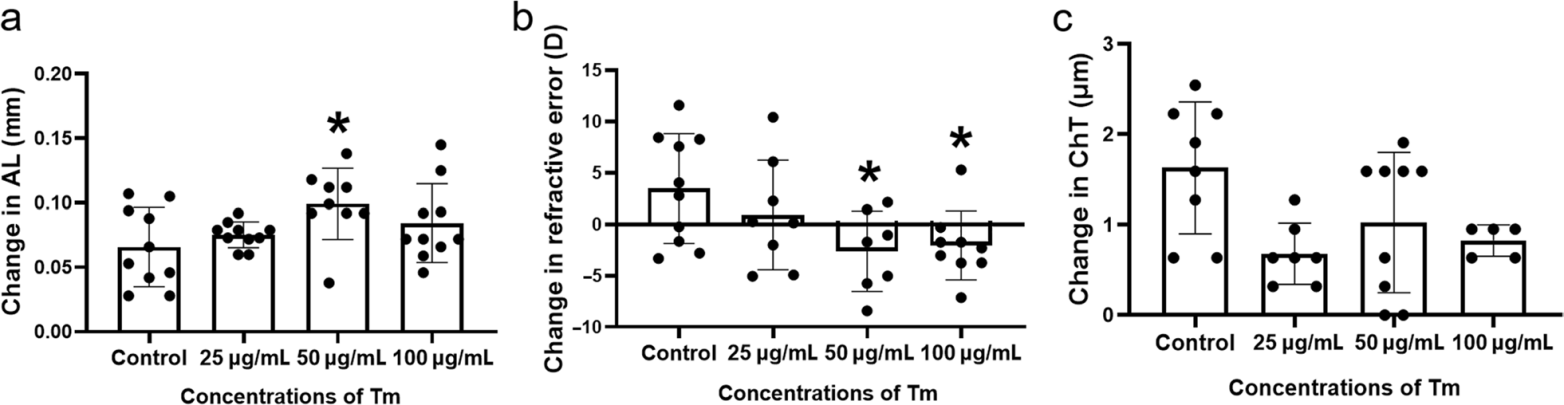
Myopic changes in mice after treatment with different concentrations of tunicamycin (Tm) eyedrops. **a** Changes in axial length (AL) at different concentrations of Tm. **b** Changes in refraction at different concentrations of Tm. **c** Changes in choroidal thickness (ChT) at different concentrations of Tm (n = 10 eyes/group, mice with missing data are excluded). Statistically significant differences are shown [compared with control group, *P < 0.05, one-way ANOVA with least significant difference (LSD) post hoc test]

To determine the appropriate concentration of Tm for inducing myopia, we initiated topical application of three different concentrations to the mice using eyedrops once and measured the refraction, axial length (AL), and the ChT before and one week after Tm administration. Mice treated with eyedrops containing 50 μg/mL Tm showed the highest AL elongation (Fig. 1a) and refractive shift towards myopia (Fig. 1b). However, there was no statistically significant difference in the change in ChT between the groups (Fig. 1c). These results suggest that Tm (50 mg/mL) is adequate for inducing myopia.

### A single dose of Tm was sufficient to induce myopia in C57BL/6 J mice

**Fig. 2:**
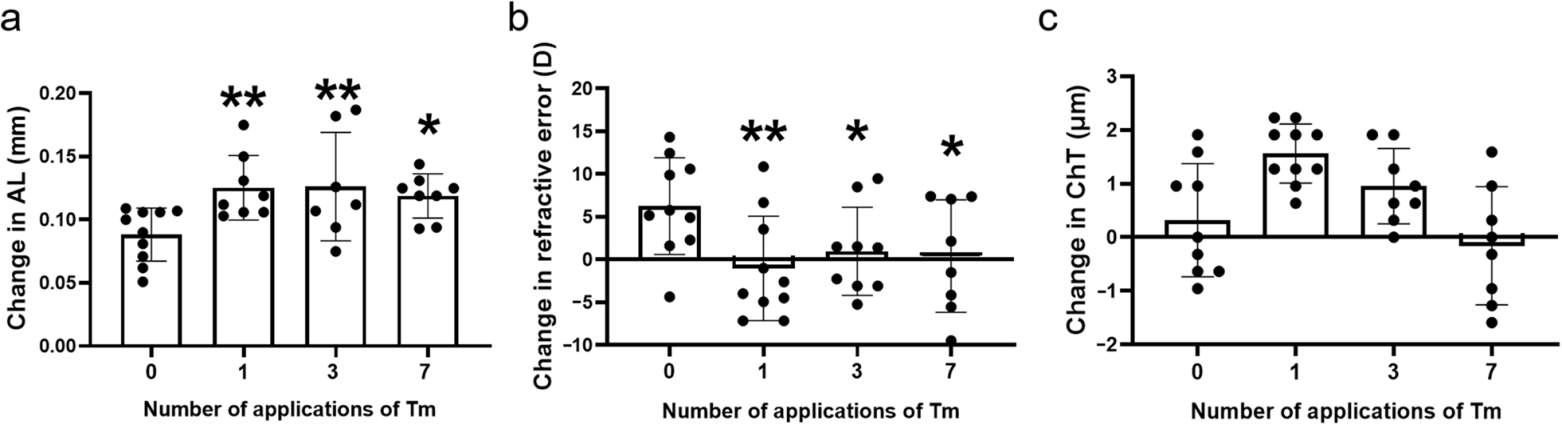
Myopia changes in mice according to number of applications of tunicamycin (Tm) eyedrops. **a** Changes in axial length (AL) according to the number of applications of Tm. **b** Changes in refraction according to the number of applications of Tm. **c** Changes in choroidal thickness (ChT) according to the number of applications of Tm (n = 10 eyes/group, mice with missing data are excluded). Statistically significant differences are shown [compared with control group, *P < 0.05, **P < 0.01, one-way ANOVA with least significant difference (LSD) post hoc test]

The optimal concentration chosen for subsequent treatments was 50 μg/mL. To identify the optimum number of applications, the effects of Tm according to the number of applications were assessed. Compared with the control group, Tm caused a higher elongation of AL, irrespective of the number of applications (Fig. 2a). Nevertheless, regarding refractive changes, the most statistically significant differences compared to the control group were observed with only a single dose of Tm (Fig. 2b). No difference was observed in the change in ChT between the control and Tm groups, regardless of the number of applications (Fig. 2c). To study the long-term effect of Tm, we monitored AL three weeks after application of a single dose of Tm. As shown in Additional file 1: Fig. S2a, there were no differences in AL at baseline between the two groups. Compared with the control group, the AL in the Tm groups was significantly higher starting in the first week, and this difference was sustained throughout the three weeks (Additional file 1: Fig. S2b). Furthermore, AL of the “once-per-week Tm eye drop” group over three weeks (TTT group) was comparable with that of the “only once Tm eye drop” group (Additional file 1: Fig. S1d, 2c, 2d). The results further highlight that a single Tm eye drop is sufficient to induce myopia in C57BL/6 J mice.

### Instillation of Tm altered the expression and organization of scleral collagen in C57BL/6 J mice

**Fig. 3:**
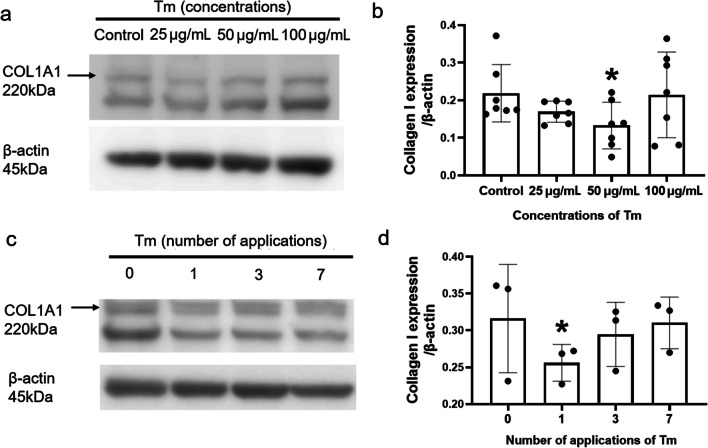
Scleral collagen concentration in scleral tissues measured using Western blotting. **a** Protein expression level of COL1A1 at different concentrations of tunicamycin (Tm). **b** Densitometric analysis of COL1A1 at different concentrations of Tm (each group n = 7; each sample was a pool of three sclerae). **c** Protein expression level of COL1A1 according to the number of applications of Tm. **d** Densitometric analysis of COL1A1 according to the number of applications of Tm (each group n = 3; each sample was a pool of three sclerae). Statistically significant differences are shown [compared with control group, *P < 0.05, one-way ANOVA with least significant difference (LSD) post hoc test]

**Fig. 4:**
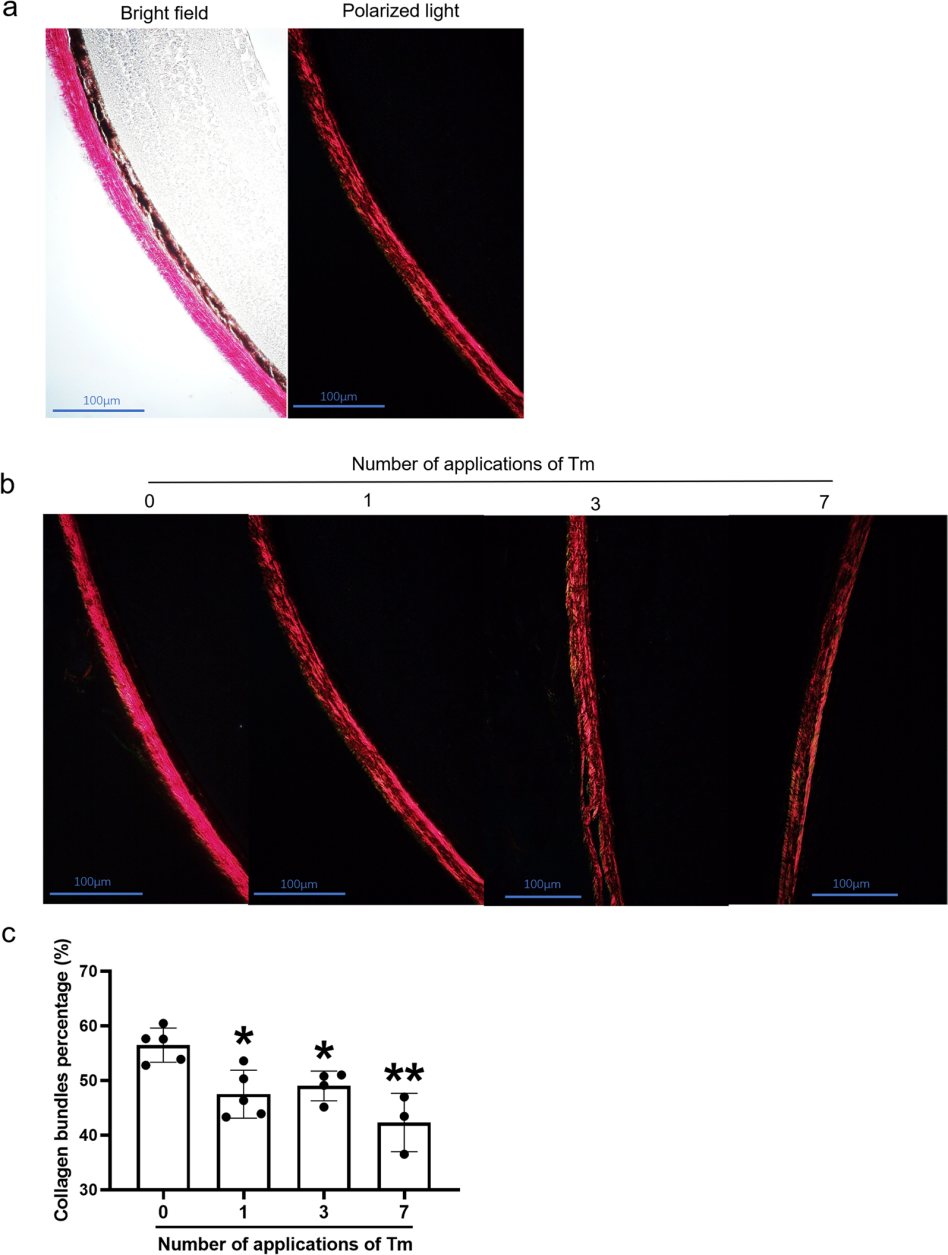
Organization of scleral collagen visualized under a microscope by picrosirius red staining. **a** Scleral collagen visualized under bright field and polarized light. **b** Organization of scleral collagen after varying number of tunicamycin (Tm) applications. **c** Concentration of collagen bundles in the sclera after different number of applications of Tm (the collagen volume fraction: ratio of collagen bundles to total collagen, n = 5 eyes/group, mice with missing data are excluded). Statistically significant differences are shown [compared with control group, *P < 0.05, **P < 0.01, one-way ANOVA with least significant difference (LSD) post hoc test]

Collagen is the major component of the ECM in various human tissues and contributes to the maintenance of the structure and strength of tissues. This study investigated the effects of various concentrations and number of applications of Tm on collagen I protein expression in the sclera using Western blotting. Treatment with a single dose of an intermediate concentration (50 μg/mL) of Tm led to the lowest expression of collagen I in the scleral tissue (Fig. 3). Not only is the amount of collagen important but also the orientation or alignment of collagen which is important for maintaining tissue structure. Therefore, we used picrosirius red staining to analyse collagen morphology in the sclera. Tm treatment caused morphological changes in collagen, which became sparse and unbundled (Fig. 4a and b). A significantly lower percentage of bundled collagen was observed in the Tm group (Fig. 4c).

### ER stress may be involved in the induction of myopia with Tm

**Fig. 5:**
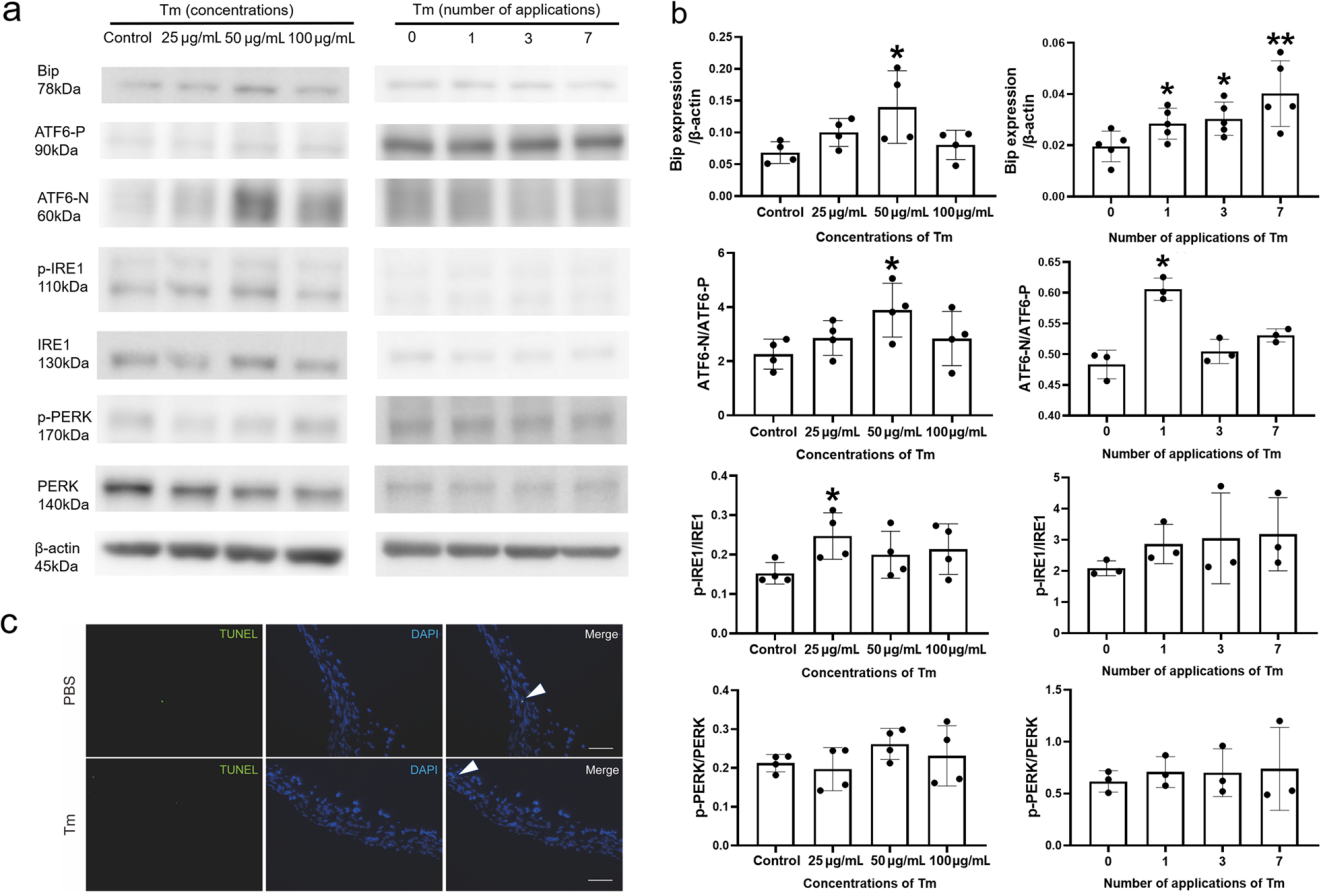
Scleral endoplasmic reticulum (ER) stress and apoptosis expression one week after treatment with tunicamycin (Tm) were detected by Western blotting and TUNEL assay, respectively. **a** Representative Western blotting results. **b** Densitometric analysis of blots in (**a**) in different groups. In each group of different Tm concentrations, n = 4 (each sample was a pool of three sclerae). In each group of different Tm applications, n = 3 (each sample was a pool of three sclerae). **c** TUNEL images (green: TUNEL, blue: DAPI) of sclerae in C57BL/6 J mice. Arrowheads indicate TUNEL-positive nuclei. Scale bar: 50 µm. Statistically significant differences are shown [*P < 0.05, one-way ANOVA with least significant difference (LSD) post hoc test]

Tm is a commonly used ER stress inducer; therefore, we determined ER stress-related protein levels in the scleral tissue. Figures 5a and b showed an increase in Bip expression, which suggested that the sclerae in the Tm group were under ER stress. To determine which unfolded protein response signal was activated, we further probed for the three classical pathway proteins and found that the activating-transcription factor 6 (ATF6) and inositol-requiring enzyme 1 (IRE1) pathways were significantly activated with Tm treatment. Although the results showed an upward trend for phospho-protein kinase RNA-like ER kinase (p-PERK) the increase was not statistically significant. TUNEL analysis did not show any change in scleral tissue in mice between the Tm and control groups (Fig. 5c). Further studies demonstrated that administration with Tm only induced ER stress on scleral tissue and did not affect other tissues of the eye (Additional file 1: Fig. S6). Our results suggest that using Tm eyedrops to induce myopia can lead to ER stress in the sclera but not apoptosis.

### Tm may regulate ECM remodelling through the promotion of collagen degradation via MMP2

**Fig. 6:**
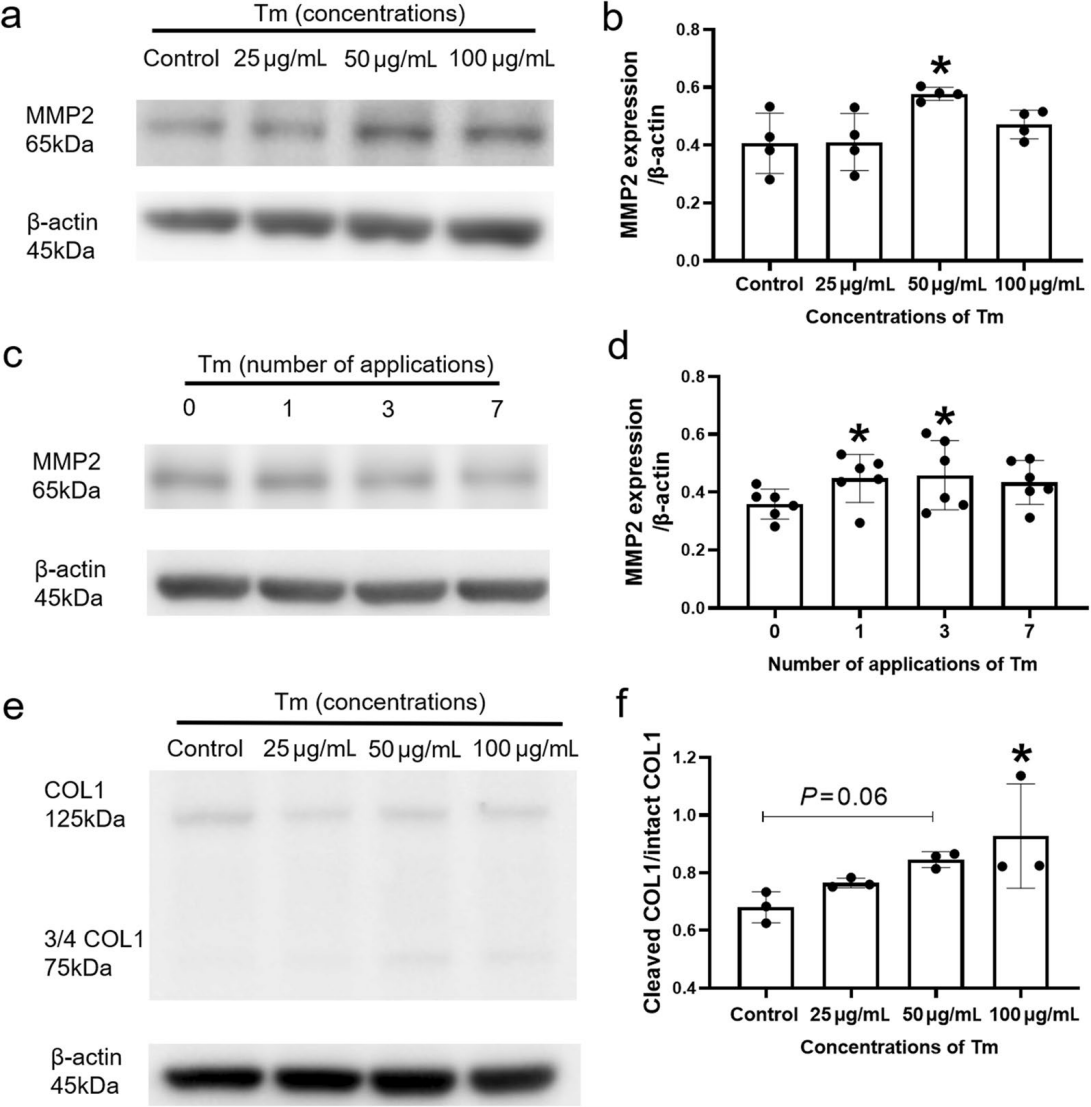
Expression levels of matrix metalloproteinase-2 (MMP2) and cleaved COL1 in sclera tissues detected using Western blotting. **a** Protein expression level of MMP2 at different tunicamycin (Tm) concentrations. **b** Densitometric analysis of MMP2 at different Tm concentrations (each group n = 4, each sample was a pool of three sclerae). **c** Protein expression level of MMP2 according to the number of Tm applications. **d** Densitometric analysis of MMP2 according to number of Tm applications (each group n = 6, each sample was a pool of three sclerae). **e** Protein expression level of cleaved COL1 (3/4 COL1) and intact COL1 in different concentrations of applications of Tm. **f** Densitometric analysis of the ratio of cleaved COL1 and intact COL1 at different Tm concentrations (each group n = 3, each sample was a pool of three sclerae). Statistically significant differences are shown [compared with control group, *P < 0.05, one-way ANOVA with least significant difference (LSD) post hoc test]

MMP2 appears to be closely related to myopia and degradation of ECM. Therefore, we evaluated the expression of MMP2 protein in the sclera and found that treatment with 50 μg/mL Tm led to a remarkable increase in MMP2 (Fig. 6a and b). Our data indicated that both a single dose and three consecutive days of Tm administration could increase the expression of MMP2 (Fig. 6c and d). To assess collagen degradation, we used an antibody which can recognise a neoepitope at the 1/4–3/4 collagen cleavage site and found that treatment with Tm increased collagen cleavage (Fig. 6e).

## Discussion

Myopia is a global ocular disease. Implementing a suitable animal model is crucial for the investigation of disease pathogenesis and efficacy of therapeutic intervention strategies. Currently, the most widely used animal models in myopia research are LIM and FDM. However, these two models have disadvantages such as invasiveness and complicated procedures. Therefore, there is a need to explore a new, effective, and simple animal model for myopia research.

Myopia induction in mice by LIM requires at least three weeks of induction. For example, wearing a negative lens with a power of − 30 D for three weeks results in a myopic shift in refraction of − 10 to − 30 D with an axial elongation of 50–60 μm [2, 14, 15]. Induction of myopia in mice by FDM takes time similar to LIM, and after four weeks of induction, a myopic shift in refraction of − 6 D to − 10 D and an elongation of the ocular axis of about 50 μm occurs [16, 17]. In our Tm-induction model, a relatively stable myopic performance was achieved at only one week after treatment with Tm eye drops, resulting in a 6–8 D refractive change (control 3.462 ± 5.335 D, Tm − 2.634 ± 3.900 D) and about 30 μm difference in axial change compared with the control group (control 0.0657 ± 0.031 mm, Tm 0.0992 ± 0.027 mm). For researchers, our model induces myopia quicker, is non-invasive and easier to administer, and thus more suitable than LIM and FDM for the study of myopia. However, the new Tm-induction model should be used with caution as it did not show the choroidal thinning that is characteristic in LIM or in humans with high myopia [18–20].

The ER is an important organelle for protein synthesis and plays a pivotal role in cellular function and cell fate determination. Excessive accumulation of misfolded proteins in the ER may induce ER stress and a series of responses called the unfolded protein response, which is emerging as a key contributor to many human diseases, including cancer, inflammatory diseases, and immune-related diseases [21–23]. ER stress has been demonstrated to be closely associated with ECM remodelling in various diseases, and is likely to be associated with the occurrence and development of myopia [24, 25]. Our previous results demonstrated that scleral ER stress plays a crucial role in the development of myopia [6]. Thus, it is reasonable to assume that myopia can be developed by inducing ER stress in the sclera.

In this study, as a classic ER stress inducer, Tm was used to induce myopia in mice using eyedrops. As shown in Figs. 1 and 2, Tm administration induced phenotypes of myopia, such as AL elongation and myopic shift in refraction, and that an appropriate concentration was required to induce these phenotypes. Moreover, a single Tm administration was sufficient to induce myopia, and the effect lasted for three weeks (Additional file 1: Fig. S2), which suggests that the effect of Tm is long-lasting. The pharmacokinetics and pharmacodynamics of antibiotics are influenced by several factors, such as pH, concentration and so on [26]. In some specific cases, some antibiotics show an effective concentration of a reverse “U”-type performance. For P. aeruginosa, high concentrations of amoxicillin were less effective. The same condition occurred for cefotaxime on E. coli [27]. Similarly, Tm as a nucleoside antibiotic, also appeared to be in a non-dose-dependent manner in the present study. At the same time, Tm is also a classic ER stress inducer. Clay et al. found that for yeast cells, low concentrations of Tm increased the load of misfolded proteins, but not high concentration [28]. Armengol et al. showed that 5 μg/mL Tm was the best concentration for inducing ER stress in HepG2 cells [29]. Others showed that α-SMA^+^ active scleral fibroblasts seem to play an important role in the progression of myopia [30] while excessive ER stress results in apoptosis. Consequently, a suitable concentration which could induce ER stress without inducing apoptosis may be the optimal concentration. As Tm eye drops are applied to the ocular surface, its effect on the cornea is also considered. We compared the measurement of the corneal curvature and found that there was no significant difference between the control and Tm groups (Additional file 1: Fig. S2e). The surface area of the conjunctiva is 17 times bigger than that of the cornea and the permeability can be as high as 29 times that of the cornea, and thus Tm can enoughly affect the sclera through the conjunctiva-sclera pathway [31]. From the above results, we considered that the effect of the drug on the cornea is limited. Additional experiments were performed to evaluate which tissues were affected by Tm by enucleating the eye 1 h and 24 h after Tm eyedrops and detected ER stress in the cornea, lens, retina, choroid, and sclera. The results showed an increase in the active form of ATF6 in the sclera at 1 h after Tm eye drops and an increase in p-eIF2 levels and the active form of ATF6 at 24 h, which were not observed in other tissues (Additional file 1: Fig. S6).

Active scleral ECM remodelling has been reported in several myopia models [9, 32, 33]. The scleral ECM contains collagen, elastin, and paraproteins, of which, collagen functions primarily as a scaffold of the ECM [34]. Collagen forms large fibrils and complex fibrous superstructures in tissues, which are primarily responsible for their tensile strength [35]. Therefore, the ECM of the sclera of myopic eyes may be abnormally weak, resulting in larger scleral system deformations. Notably, our results show that Tm instillation may cause marked alterations in scleral collagen. The expression of the COL1A1 protein in scleral tissues decreased (Fig. 3). Furthermore, we performed visual observations of the sclera under polarised light to determine the collagen structure. Collagen fibres became scattered and unbundled when treated with Tm (Fig. 4). Since IOP is important for the development of myopia, we measured IOP after treatment with Tm and the results showed that Tm eye drops did not influence IOP (Additional file 1: Fig. S2f). Hence, ER stress could induce myopia i.e., scleral ER stress may contribute to AL elongation by making the collagen assembly loose and easily deformable.

Scleral ECM is maintained by a dynamic balance between protein synthesis and degradation. MMP2, an interstitial collagenase, can cleave soluble collagen I into 3/4- and 1/4-length fragments [36]. Previous studies have shown that MMP2 is expressed in the myopic sclera and plays a role in scleral remodelling [37–39]. In the FDM model of tree shrews, an exogenous tissue inhibitor of metalloproteinase 2, which is an endogenous inhibitor of MMP2, inhibited the development of myopia [40]. A similar situation occurred in LIM tree shrews; when myopia is induced, the mRNA level of MMP2 increases significantly and subsequently decreases in the recovery period [41]. Thus, we investigated the expression of MMP2 in the sclera and found that Tm administration upregulated the expression of MMP2 in the sclera and, in turn, resulted in an increase in cleaved collagen I protein expression (Fig. 6). A significant association was observed between MMP2 and ER stress. For example, in a mouse model of glaucoma, reducing ER stress could reduce intraocular pressure by inhibiting the activity of MMP2 [42]. Similarly, ATF4, which is downstream of PERK, regulated the expression of MMP2 in a mouse model of oesophageal squamous cell carcinoma [43]. We speculated that the change in collagen levels in our model may be due to increased collagen degradation by MMP2.

As a model of chemically induced myopia in mice, our study has some shortcomings such as the fact that we did not examine Tm concentrations in other parts of the eye (layers of the eyeball wall and eye contents). Based on the shortcomings of this research, we will conduct a more in-depth study of this model to optimize its use for myopia research.

## Conclusions

Tm eyedrops induced myopic changes, loosening of the scleral collagen, and decreased expression of collagen I. This process may be associated with ER stress in the sclera. We not only developed a convenient and promising addition to existing myopia models for future research, but also revealed a possible mechanism of myopia development. Finally, the findings were limited to the eyes of C57BL/6 J mice and further research will be required to refine this model in the future.

### Supplementary Information

**Additional file 1: Figure S1.** Experimental plan for animals. **a** In the different concentrations of tunicamycin (Tm) experiment, refraction and axial length (AL) were evaluated on day 0 and day 7, and the mice were treated with Tm or dimethyl sulfoxide (DMSO) eyedrops. **b** In the different number of Tm applications experiment, refraction and AL were evaluated on day 0 and day 7; the single-application group was treated with Tm on day 0, the three-application group was treated with Tm on the first three days, the seven-application group was treated with Tm every day, and mice in the control group were treated with the same concentration of DMSO on day 0, and received phosphate-buffered saline (PBS) on other days. **c** In the long-term experiment, refraction and AL were evaluated on days 0, 7, and 21, and mice were treated with Tm or DMSO eyedrops on day 0. **d** In the long-term different number of Tm applications experiment, AL were evaluated on day 0 and day 21; the PPP group (PBS-PBS-PBS) was treated with PBS on days 0, 7 and 14, the TPP group (Tm-PBS-PBS) was treated with Tm on day 0 and treated with PBS on days 7 and 14, the TTT group (Tm–Tm-Tm) was treated with Tm on days 0, 7 and 14. Finally, the mice were euthanised, eyeballs were harvested for paraffin sectioning, and the sclerae were collected to extract proteins for Western blotting.

**Additional file 2: Figure S2.** Long-term effect of tunicamycin (Tm) eyedrops on myopia induction. **a** Changes in axial length (AL) over time. **b** Changes in AL at one week and three weeks after treatment with Tm (n = 10 eyes/group, mice with missing data are excluded). **c** Changes in AL over time during different number of Tm applications experiment. **d** Changes in AL at three weeks after treatment with Tm or phosphate-buffered saline (PBS) (PPP group means PBS-PBS-PBS, which were treated with PBS on days 0, 7 and 14, n = 6 eyes/group; TPP group means Tm-PBS-PBS, which were treated with Tm on day 0 and treated with PBS on days 7 and 14, n = 8 eyes/group; TTT group means Tm–Tm-Tm, which were treated with Tm on days 0, 7 and 14, n = 8 eyes/group). **e** Changes in corneal curvature at 1, 2 and 3 weeks (n = 10 eyes/group, mice with missing data are excluded). f Changes in intraocular pressure at one week after treatment with Tm (n = 8 eyes/group). Statistically significant differences are shown (compared to the control group, ∗ *P* < 0.05, ∗∗ *P* < 0.01, ns, no significant difference, Student’s t-test).

**Additional file 3: Figure S3.** Uncropped blots in this manuscript (Figs. 3, 4, 5, 6). **a** Uncropped blots in Fig. 3a. **b** Uncropped blots in Fig. 3c. **c** Uncropped blots in Fig. 5a (left). **d** Uncropped blots in Fig. 5a (right). **e** Uncropped blots in Fig. 6a. **f** Uncropped blots in Fig. 6c. **g** Uncropped blots in Fig. 6e.

**Additional file 4: Figure S4.** Bundled collagen analysis was performed using Image J (v.1.53, NIH) using the threshold method. **a** Scleral collagen visualized under bright field. **b** Scleral collagen visualized under polarized light. **c** All reconstructed images were converted to 8-bit. **d** Bundled collagen fibres are shown by setting a threshold. **e** Unbundled collagen fibres are also shown by setting a threshold.

**Additional file 5: Figure S5.** Images of ocular components measurement. **a** Image of axial length measurement. **b** Image of refraction measurement. c Image of choroidal thickness measurement. **d** Image of corneal curvature measurement.

**Additional file 6: Figure S6.** Expression of ATF6, p-eIF2 and eIF2 in the different eye tissues were detected using Western blotting. **a** Expression levels of ATF6, p-eIF2 and eIF2 in the sclera. **b** Expression levels of ATF6, p-eIF2 and eIF2 in the cornea. **c** Expression levels of ATF6, p-eIF2 and eIF2 in the lens. **d** Expression levels of ATF6, p-eIF2 and eIF2 in the retina. **e** Expression levels of ATF6, p-eIF2 and eIF2 in the choroid.

## Data Availability

The data used to support the findings of this study are available from the corresponding author upon request.

## Abbreviations

ER: Endoplasmic reticulum
Tm: Tunicamycin
AL: Axial length
IOP: Intraocular pressure
MMP2: Matrix metalloproteinase-2
FDM: Form-deprivation myopia
LIM: Lens-induced myopia
ECM: Extracellular matrix
ChT: Choroidal thickness
ATF6: Activating-transcription factor 6
IRE1: Inositol-requiring enzyme 1
p-PERK: Phospho-protein kinase RNA-like ER kinase
DMSO: Dimethyl sulfoxide
PBS: Phosphate-buffered saline
SD-OCT: Spectral domain optical coherence tomography
DI: Deionised water
LSD: Least significant difference
ANOVA: Analysis of variance
