# Supplementary figures and images for: Establishment of a novel ER-stress induced myopia model in mice
Source: Eye Vis (Lond). 2023 Nov 1;10:44. doi: 10.1186/s40662-023-00361-2 (PMC10619234; doi:10.1186/s40662-023-00361-2)

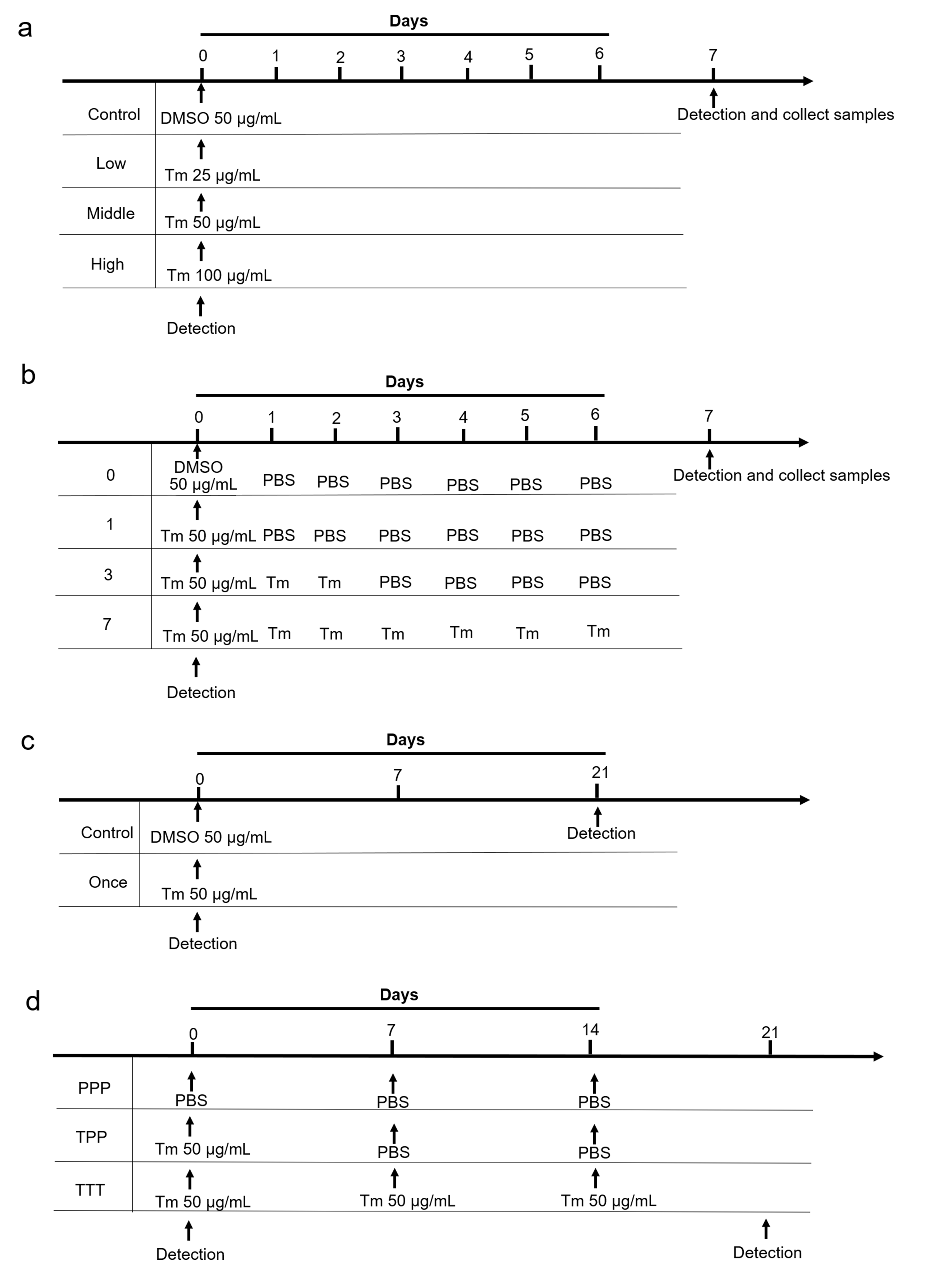

Supplement: Supplementary file 1 — Additional file 1: Figure S1. Experimental plan for animals. a In the different concentrations of tunicamycin (Tm) experiment, refraction and axial length (AL) were evaluated on day 0 and day 7, and the mice were treated with Tm or dimethyl sulfoxide (DMSO) eyedrops. b In the different number of Tm applications experiment, refraction and AL were evaluated on day 0 and day 7; the single-application group was treated with Tm on day 0, the three-application group was treated with Tm on the first three days, the seven-application group was treated with Tm every day, and mice in the control group were treated with the same concentration of DMSO on day 0, and received phosphate-buffered saline (PBS) on other days. c In the long-term experiment, refraction and AL were evaluated on days 0, 7, and 21, and mice were treated with Tm or DMSO eyedrops on day 0. d In the long-term different number of Tm applications experiment, AL were evaluated on day 0 and day 21; the PPP group (PBS-PBS-PBS) was treated with PBS on days 0, 7 and 14, the TPP group (Tm-PBS-PBS) was treated with Tm on day 0 and treated with PBS on days 7 and 14, the TTT group (Tm–Tm-Tm) was treated with Tm on days 0, 7 and 14. Finally, the mice were euthanised, eyeballs were harvested for paraffin sectioning, and the sclerae were collected to extract proteins for Western blotting. [file 40662_2023_361_MOESM1_ESM.png]

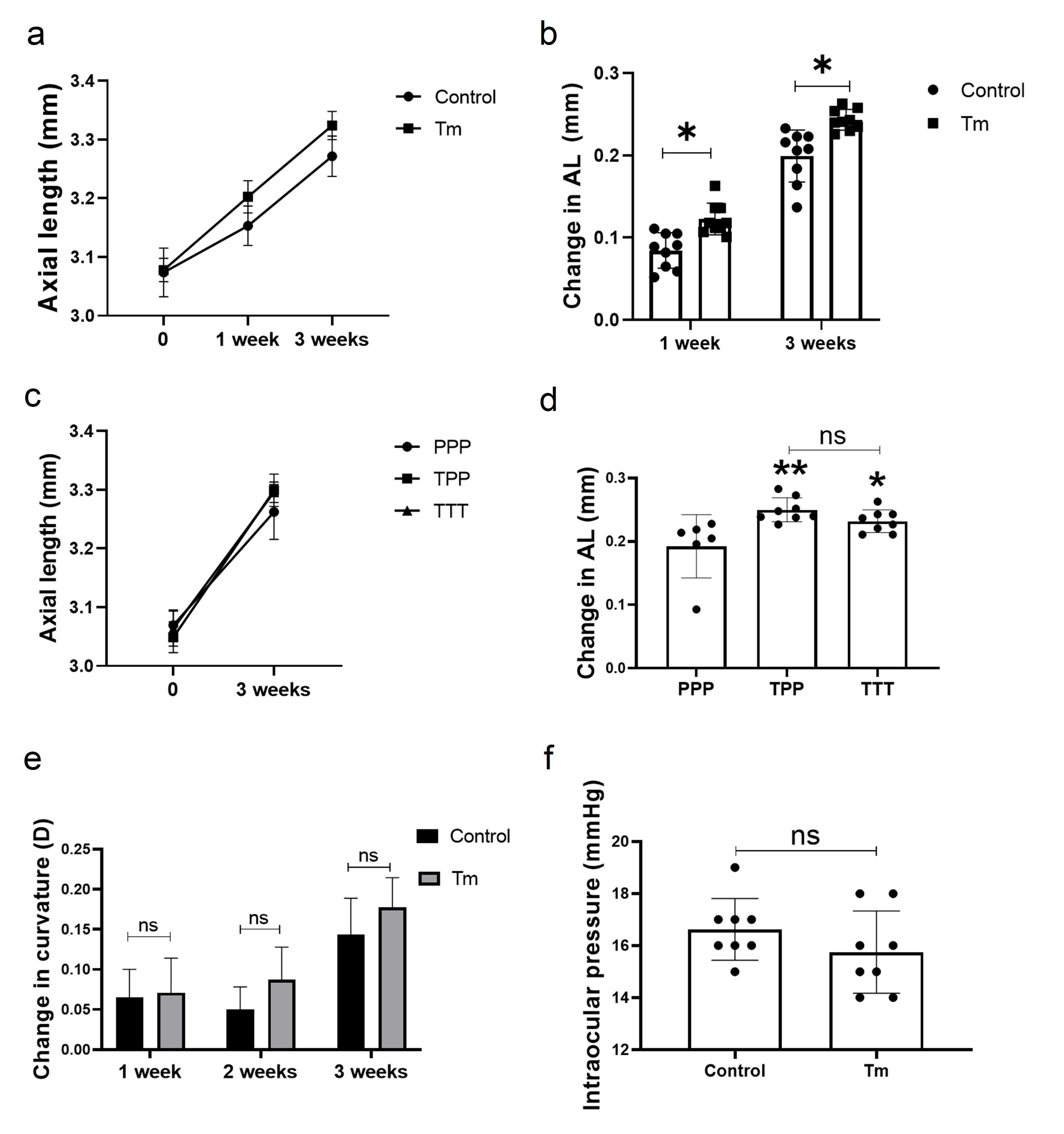

Supplement: Supplementary file 2 — Additional file 2: Figure S2. Long-term effect of tunicamycin (Tm) eyedrops on myopia induction. a Changes in axial length (AL) over time. b Changes in AL at one week and three weeks after treatment with Tm (n = 10 eyes/group, mice with missing data are excluded). c Changes in AL over time during different number of Tm applications experiment. d Changes in AL at three weeks after treatment with Tm or phosphate-buffered saline (PBS) (PPP group means PBS-PBS-PBS, which were treated with PBS on days 0, 7 and 14, n = 6 eyes/group; TPP group means Tm-PBS-PBS, which were treated with Tm on day 0 and treated with PBS on days 7 and 14, n = 8 eyes/group; TTT group means Tm–Tm-Tm, which were treated with Tm on days 0, 7 and 14, n = 8 eyes/group). e Changes in corneal curvature at 1, 2 and 3 weeks (n = 10 eyes/group, mice with missing data are excluded). f Changes in intraocular pressure at one week after treatment with Tm (n = 8 eyes/group). Statistically significant differences are shown (compared to the control group, ∗ P < 0.05, ∗∗ P < 0.01, ns, no significant difference, Student’s t-test). [file 40662_2023_361_MOESM2_ESM.png]

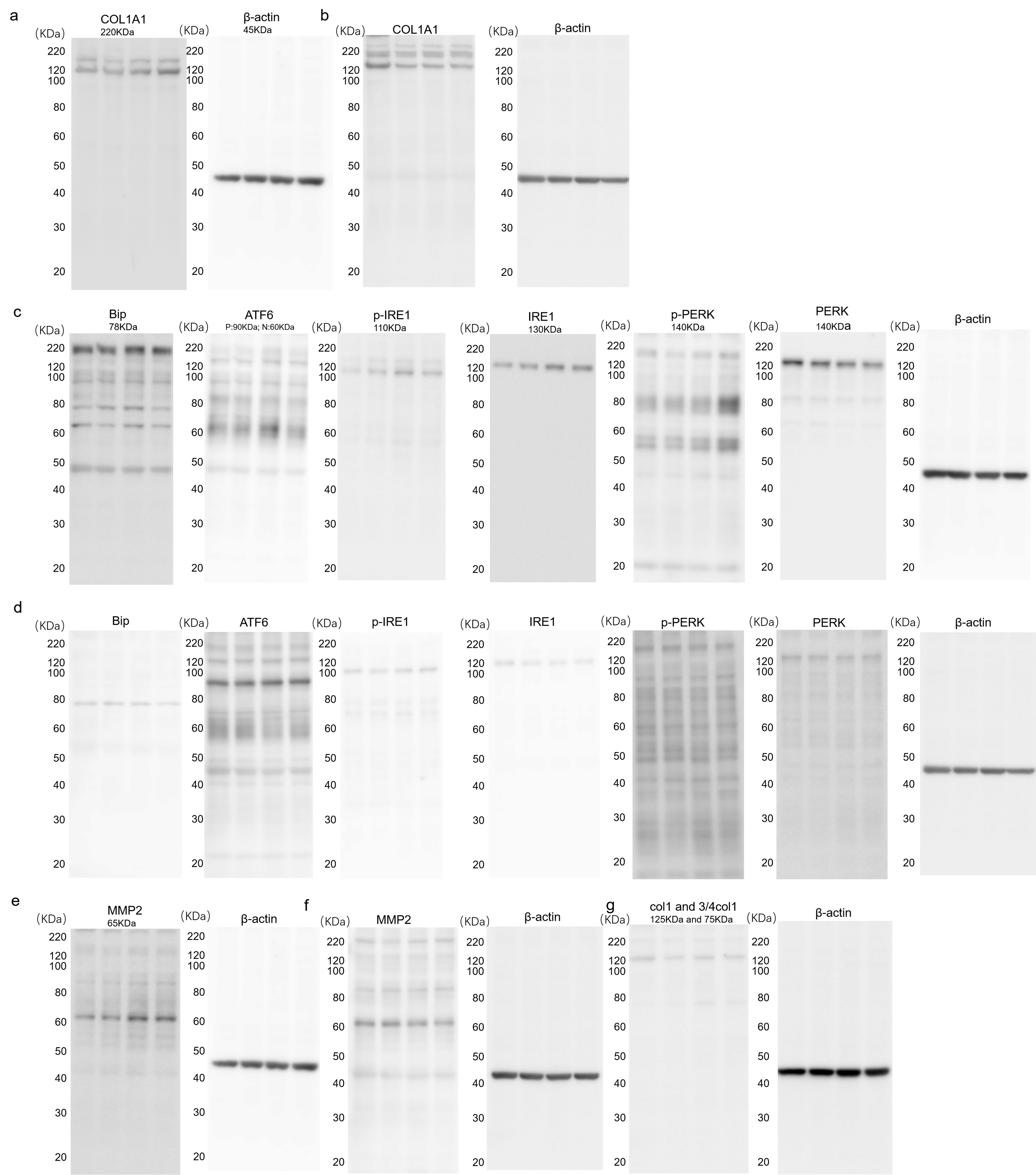

Supplement: Supplementary file 3 — Additional file 3: Figure S3. Uncropped blots in this manuscript (Figs. 3, 4, 5, 6). a Uncropped blots in Fig. 3a. b Uncropped blots in Fig. 3c. c Uncropped blots in Fig. 5a (left). d Uncropped blots in Fig. 5a (right). e Uncropped blots in Fig. 6a. f Uncropped blots in Fig. 6c. g Uncropped blots in Fig. 6e. [file 40662_2023_361_MOESM3_ESM.png]

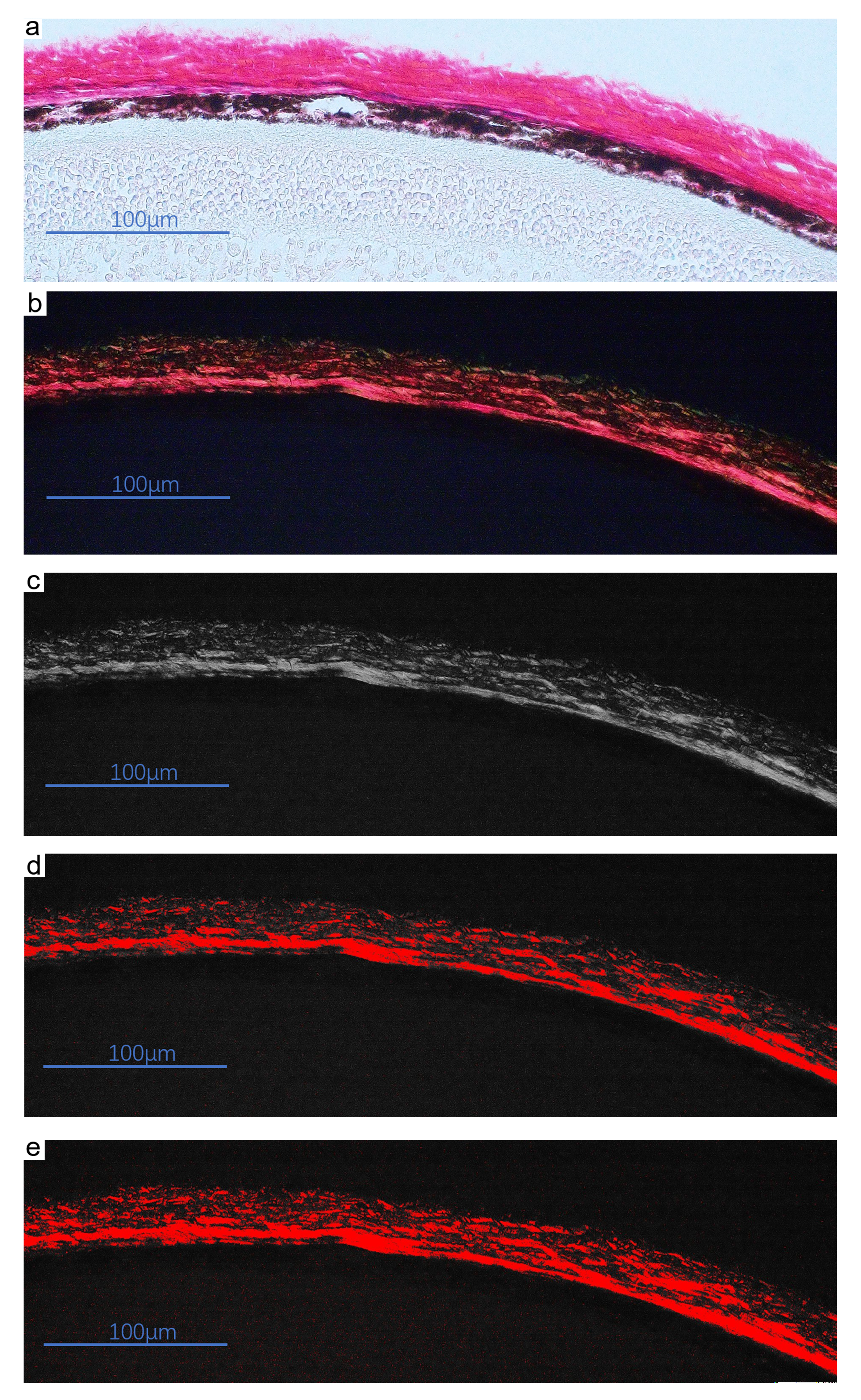

Supplement: Supplementary file 4 — Additional file 4: Figure S4. Bundled collagen analysis was performed using Image J (v.1.53, NIH) using the threshold method. a Scleral collagen visualized under bright field. b Scleral collagen visualized under polarized light. c All reconstructed images were converted to 8-bit. d Bundled collagen fibres are shown by setting a threshold. e Unbundled collagen fibres are also shown by setting a threshold. [file 40662_2023_361_MOESM4_ESM.png]

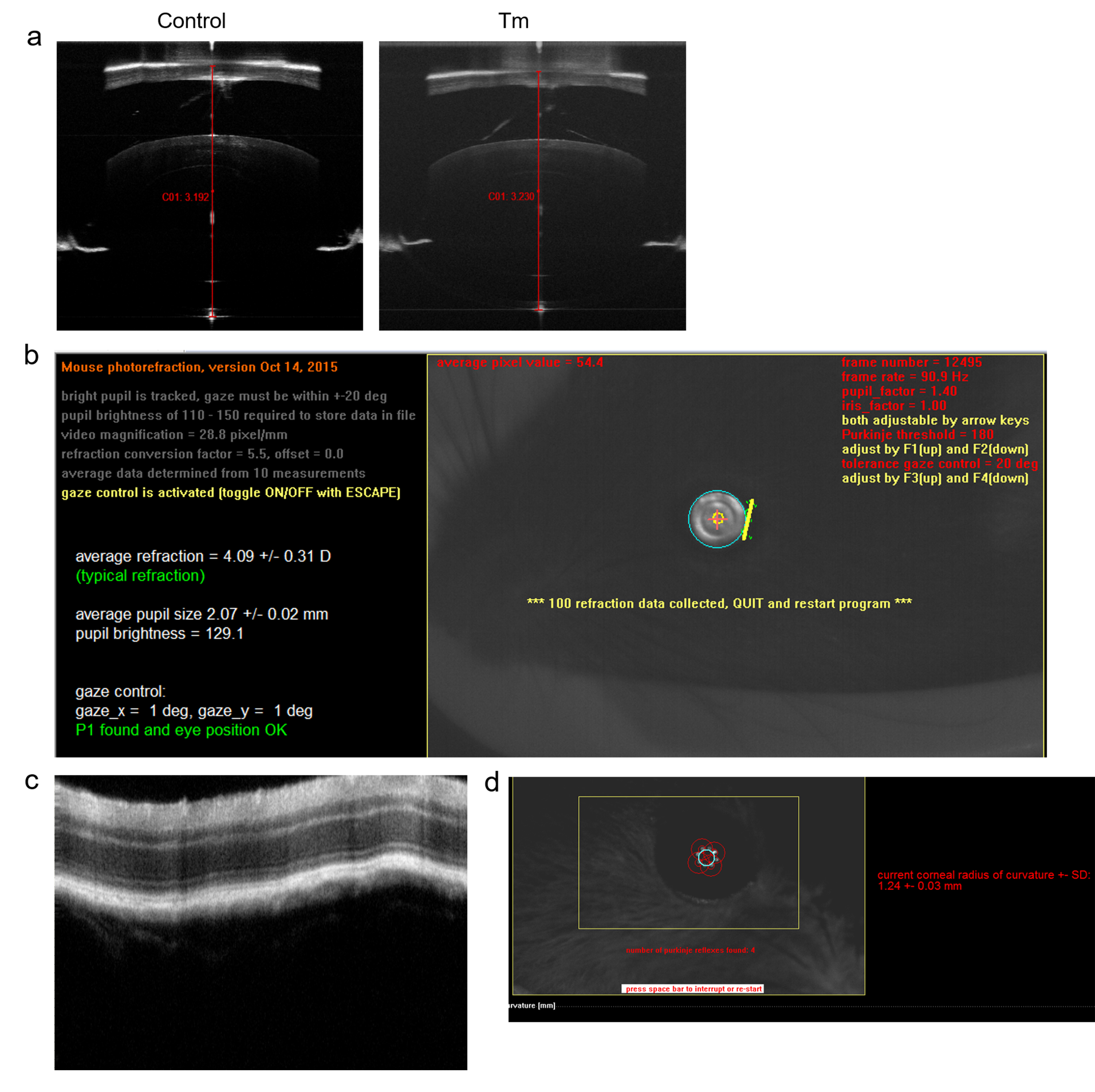

Supplement: Supplementary file 5 — Additional file 5: Figure S5. Images of ocular components measurement. a Image of axial length measurement. b Image of refraction measurement. c Image of choroidal thickness measurement. d Image of corneal curvature measurement. [file 40662_2023_361_MOESM5_ESM.png]

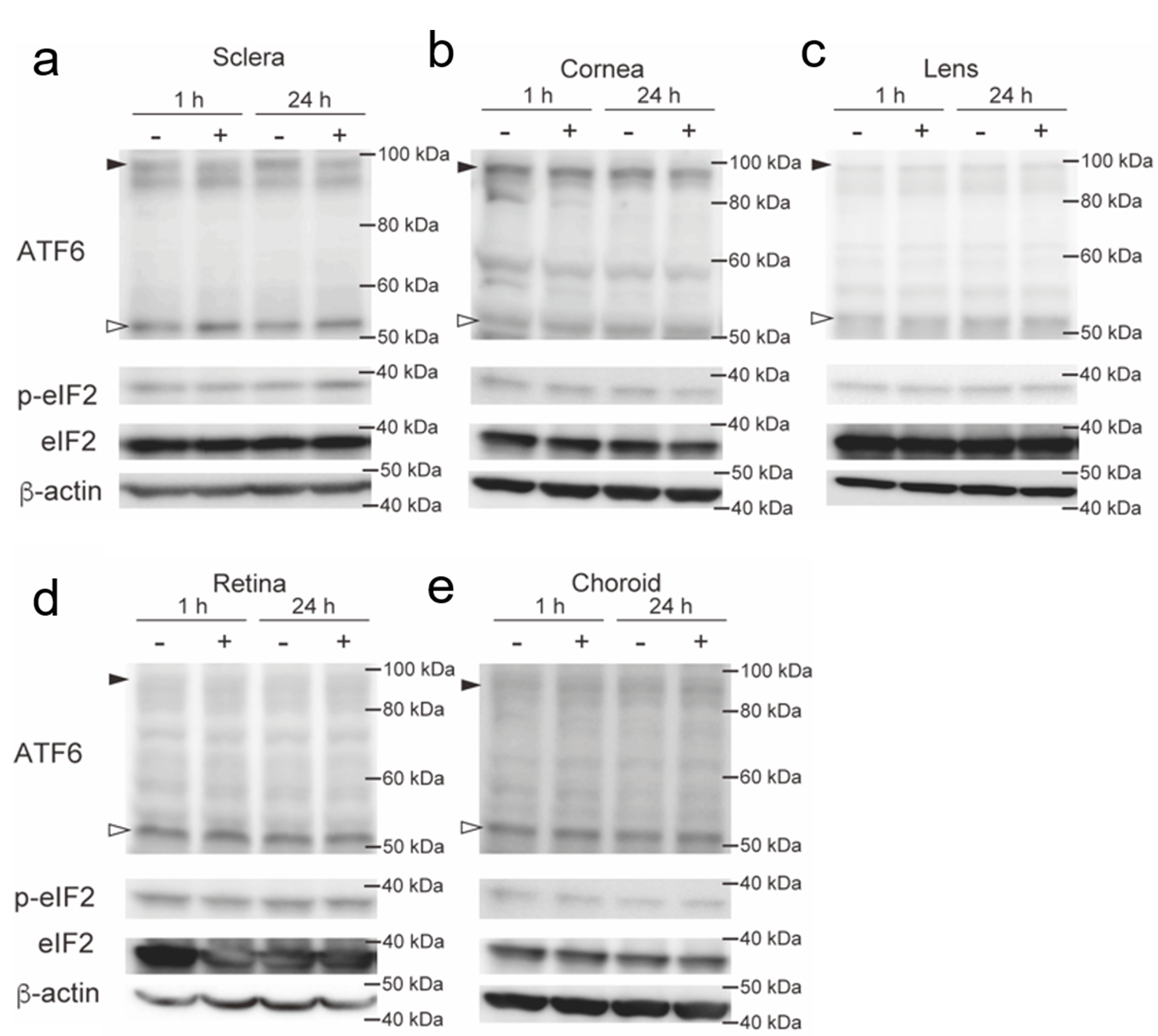

Supplement: Supplementary file 6 — Additional file 6: Figure S6. Expression of ATF6, p-eIF2 and eIF2 in the different eye tissues were detected using Western blotting. a Expression levels of ATF6, p-eIF2 and eIF2 in the sclera. b Expression levels of ATF6, p-eIF2 and eIF2 in the cornea. c Expression levels of ATF6, p-eIF2 and eIF2 in the lens. d Expression levels of ATF6, p-eIF2 and eIF2 in the retina. e Expression levels of ATF6, p-eIF2 and eIF2 in the choroid. [file 40662_2023_361_MOESM6_ESM.png]
